# Morphological, chemical and species delimitation analyses provide new taxonomic insights into two groups of *Rinodina*

**DOI:** 10.1017/S0024282916000359

**Published:** 2016-09

**Authors:** Philipp Resl, Helmut Mayrhofer, Stephen R. Clayden, Toby Spribille, Göran Thor, Tor Tønsberg, John W. Sheard

**Affiliations:** Institute of Plant Sciences, NAWI Graz, University of Graz, Holteigasse 6, 8010 Graz, Austria; New Brunswick Museum, 277 Douglas Avenue, Saint John, New Brunswick E2K 1E5, Canada; Institute of Plant Sciences, University of Graz, Holteigasse 6, 8010 Graz, Austria; Department of Ecology, Swedish University of Agricultural Sciences, P.O. Box 7044, 750 07 Uppsala, Sweden; Department of Natural History, University Museum, University of Bergen, Allégaten 41, P.O. Box 7800, 5020 Bergen, Norway; Department of Biology, University of Saskatchewan, 112 Science Place, Saskatoon, SK S7N 5E2, Canada

**Keywords:** lichenized Ascomycetes, *Oxnerella*, *Physcia*-*Physconia*-type ascospore development, *Physciaceae*

## Abstract

The genus *Rinodina* (*Physciaceae*), with approximately 300 species, has been subject to few phylogenetic studies. Consequently taxonomic hypotheses in *Rinodina* are largely reliant on phenotypic data, while hypotheses incorporating DNA dependent methods remain to be tested. Here we investigate *Rinodina degeliana*/*R. subparieta* and the *Rinodina mniaraea* group, which previously have not been subjected to comprehensive molecular and phenotypic studies. We conducted detailed morphological, anatomical, chemical, molecular phylogenetic and species delimitation studies including 24 newly sequenced specimens. We propose that *Rinodina degeliana* and *R. subparieta* are conspecific and that chemical morphs within the *R. mniaraea* group should be recognized as distinct species. We also propose the placement of the recently described genus *Oxnerella* in *Physciaceae*.

## Introduction

Species classification and delimitation within the fungi received a strong impetus when molecular phylogenetic methods emerged (Blackwell 2011; Hibbett & Taylor 2013). Molecular methods have helped to solve several long-standing questions about higher-level taxonomic relationships among lichen-forming fungi (e.g. see James *et al.* 2006; Thell *et al.* 2012; Miadlikowska *et al.* 2014; Resl *et al.* 2015). However, applying molecular methods also creates new challenges and discussions are ongoing about how to crosslink phenotypic (including morphology, anatomy and secondary chemistry) information with DNA sequence data when drawing taxonomic conclusions such as the description of new lineages (Grube & Kroken 2000; Crespo & Lumbsch 2010; Hibbet & Taylor 2013).

The issue of reconciling molecular and phenotypic evidence to develop taxonomic hypotheses for individual species may be simplified to three different scenarios: 1) molecular and phenotypic evidence both support taxonomic conclusions; 2) taxonomic conclusions are supported by molecular but not phenotypic characters; and 3) phenotypic evidence supports taxonomic conclusions while molecular methods do not.

Not all characters may coincide with a taxonomic hypothesis. Nevertheless, a broad consensus exists that multiple independent lines of evidence in support of taxonomic conclusions should be the desired goal of every modern taxonomic study. Lichenologists, however, are often faced with the challenge of phylogenetic and phenotypic data being inconsistent (e.g. Baloch & Grube 2009; Altermann *et al.* 2014; Lücking *et al.* 2014; Singh *et al.* 2015). With more sequences becoming available, species boundaries traditionally circumscribed by phenotypic characters were challenged and it was proposed that extant taxa numbers were systematically underestimated when relying on phenotypic characters alone (Crespo & Lumbsch 2010; Lumbsch & Leavitt 2011). However, there are also cases where molecular methods have revealed that phenotypic differences might overestimate species diversity (*Xanthoparmelia*: Leavitt *et al.* 2011a, b; *Bryoria* section *Implexae*: Myllys *et al.* 2011; Velmala *et al.* 2014).

While phenotypic differences between species can be subtle (Spribille *et al.* 2014) they still represent a valuable source of information with which to formulate hypotheses on how to delimit species, especially in groups where phylogenetic data remain fragmentary. One such group, which has been subjected to rather few molecular phylogenetic studies, is the family *Physciaceae* (e.g. Lohtander *et al.* 2000, 2009; Wedin *et al.* 2000; Grube & Arup 2001; Helms *et al.* 2003; Crespo *et al.* 2004; Kaschik 2006; Nadyeina *et al.* 2010; Kondratyuk *et al.* 2014a). In this family, taxa are delimited mainly on the basis of secondary chemistry, asci and ascospores. The latter particularly have been emphasized as important and, consequently, ascospores are usually summarized into different ‘types’ (Poelt & Mayrhofer 1979; Mayrhofer & Moberg 2002; Sheard 2010). A thorough phylogenetic treatment of the *Physciaceae* including members of all known genera and sequences of multiple gene fragments to test these concepts is still missing. We focus here on two examples of closely related taxa with unclear taxonomy from the genus *Rinodina* with *Physcia*- and *Physconia*-type spores, and various secondary compounds. The combined analysis of phenotypic and molecular characters also allows us to provide integrated hypotheses of species taxonomy and nomenclatural status.

The first case was presented to us in the course of studying corticolous *Rinodina* specimens collected by GT and TT from Japan. Fertile, esorediate collections were found showing close morphological, anatomical and chemical similarities to the sorediate species *Rinodina degeliana* Coppins. *Rinodina degeliana* is scattered throughout the Northern Hemisphere. It occurs in Europe (Coppins 1983; Tønsberg 1992; Mayrhofer & Moberg 2002; Giavarini *et al.* 2009), North America (Sheard 2010; Lendemer *et al.* 2014) and Japan (GT, TT, J.W. Sheard, pers. obs.). The esorediate specimens were subsequently found to be phenotypically identical to the holotype of *Rinodina subparieta* (Nyl.) Zahlbr., a species considered to be endemic to Japan.

The treatment of otherwise identical sorediate or esorediate morphs has been discussed by Tønsberg (1992), and more recently by Brodo & Lendemer (2012), among others. For a full literature list and exhaustive discussions, please consult the references in these two papers. Suffice it to say that there is no consensus on how different morphs of lichen taxa should be recognized. Most instructive has been the molecular study of Tehler *et al.* (2009), of a single species aggregate within the genus *Roccella* where different taxa were found to be either variable or uniform with respect to their reproductive morphs. Therefore, it appears that all apparent pairs of fertile and vegetative morphs must be studied in detail in order to decide their taxonomic status. This leads us to question if the names *Rinodina degeliana* and *R. subparieta* should both continue to be recognized as distinct species distinguished by the presence or absence of soralia.

The second example is *Rinodina mniaraea* (Ach.) Körb., a terricolous taxon that has been studied in detail by Timpe (1990) and Ertl (2000). It is found in cold and moist alpine dwarf shrub and grassland habitats on bare soil, bryophytes and plant remnants (Mayrhofer & Moberg 2002; Sheard 2010). Within *R. mniaraea* several infraspecific taxa have been recognized, differing in chemistry (Mayrhofer & Moberg 2002). All these taxa regularly form apothecia with *Lecanora*-type asci, possess large *Physcia*-type ascospores and frequently contain variolaric acid. They have been accepted as varieties in a treatment of the genus *Rinodina* (Mayrhofer & Moberg 2002), although Sheard (2010) did not accept the use of varietal status. However, the names *R. cinnamomea* (Th. Fr.) Räsänen and *R. mniaraeiza* (Nyl.) Arnold already existed at the species rank for two of the varieties. How should these entities be referred to and which taxonomic rank is appropriate for them?

Here we present the results of a study using collections of *R. degeliana* and *R. subparieta* from multiple localities within their known Northern Hemisphere range and material of the three varieties of *R. mniaraea* from the European Alps and western North America. We performed detailed morphological, anatomical and chemical analyses of material from those species. Additionally, we reconstructed maximum likelihood and Bayesian phylogenies of numerous *Rinodina* taxa including *R. degeliana*, *R. subparieta*, *R. mniaraea* var. *mniaraea*, *R. mniaraea* var. *mniaraeiza* and *R. mniaraea* var. *cinnamomea* based on sequences from nuclear and mitochondrial gene fragments. We performed species delimitation methods using a Bayesian implementation of the General Mixed Yule Coalescence model (Reid & Carstens 2012) to find evidence for our two aims: 1) to examine species delimitation within these closely related sets of taxa in order to re-evaluate esorediate and sorediate morphs (in the case of *R. degeliana*/*subparieta*) and different chemotypes (*R. mniaraea* s. lat.), and 2) to decide on the status of the names *R. degeliana*, *R. subparieta* and the varieties of *R. mniaraea* s. lat.

## Materials and Methods

SC, TS, GT, TT and James Lendemer provided fresh collections for molecular analysis. For *Rinodina degeliana* and *R. subparieta*, procedures for morphological and anatomical examination, and photography, follow those of Sheard *et al.* (2014). For *R. mniaraea* s. lat., we provide revised descriptions based on Timpe (1990). Spore measurements for *R. subparieta/R*. *degeliana* are quoted as the 25%–75% range around the median with the 5% and 95% outliers in brackets. For *R. mniaraea* s. lat the average spore size and the minimum and maximum range are given. Thin-layer chromatography was carried out according to the methods of Culberson & Kristinsson (1970) and Culberson (1972) with later modifications.

As a basis for our study we selected sequences from the dataset of Nadyeina *et al.* (2010) containing a broad range of *Physciaceae* species with different ascospore types and secondary metabolites. We were especially interested in the relationships of *R. degeliana* and *R. subparieta* with other *Rinodina* species possessing *Physcia*-type ascospores and the secondary compound atranorin. In addition to the ten samples from *R. degeliana* and two from *R. subparieta*, we also included eight specimens of *R. mniaraea* from its three varieties, all possessing *Physcia*-type ascospores and sometimes atranorin. Furthermore, we sequenced *R. freyi* H. Magn. and *R. efflorescens* Malme with *Physcia*-type spores, but both lacking atranorin. To complete our dataset we included samples of the previously unsequenced or rarely sequenced species *R. trevisanii* (Hepp) Körb. and *Phaeorrhiza nimbosa* (Fr.) H. Mayrhofer & Poelt, and also the hitherto unplaced *Oxnerella safavidiorum* S. Y. Kondr. *et al*. (Table 1).

### DNA isolation, PCR amplification and sequencing

Thallus squamules or apothecia were placed in 1·5 ml Eppendorf tubes, frozen at −80 °C and ground to powder in a Retsch cell grinder. We directly applied lysis buffer to the sheared cells. DNA extraction followed the QIAmp DNA Investigator Kit protocol according to the manufacturer’s instructions. We eluted the nucleic acids in 50 μl elution buffer and used them undiluted for subsequent polymerase chain reactions (PCR). For each sample we sequenced the internal transcribed spacer regions 1 and 2 with the embedded 5.8S region of the ribosomal DNA (ITS) and the mitochondrial ribosomal small subunit (mtSSU). PCR was performed with PuReTaq Ready-To-Go PCR beads using primer pairs ITS1F/ITS4 (Gardes & Bruns 1993; White *et al.* 1990) and mtSSU1:mtSSU3R (Zoller *et al.* 1999) for ITS and mtSSU, respectively. PCR products were checked for the correct size on ethidium bromide-stained agarose gels and purified using the Omega E.Z.N.A Cycle Pure Kit according to the manufacturer’s instructions. Purified PCR products were sequenced by Microsynth (Vienna).

### Alignment and phylogenetic reconstruction and tree selection

Raw sequences were subjected to manual quality control with BioEdit 7.2.5 (Hall 1999) and only high quality sequences were used for phylogenetic analyses. Sequences were aligned and concatenated using our in-house script pipeline (Resl *et al.* 2015; Resl 2015). We aligned sequences with MAFFT v.7 (Katoh & Standley 2013) employing the –genafpair algorithm. Alignments were checked manually with BioEdit 7.2.5 and only obvious errors (when MAFFT placed single nucleotides on either end of the alignments introducing long gaps) were corrected. These alignments were then used, without excluding any sites. Data matrices and trees are deposited at Treebase.org under study ID: 19205.

We created gene trees for the two gene fragments with RAxML v.8 (Stamatakis 2014). For each gene fragment we set RAxML to generate 500 fast bootstrap replicates. We partitioned the ITS dataset into the ITS1, 5.8S and ITS2 components and used PartitionFinder 1.1.1 (Lanfear *et al*. 2012) with a greedy search strategy under a BIC criterion to determine the optimal number of partitions and substitution models for RAxML. Accordingly, the dataset was divided into a 5.8S and ITS1/ITS2 partition with GTRGAMMA respectively. For the mtSSU dataset we used one partition and the GTRGAMMA substitution model. We identified topological conflicts among gene trees with compat.py (Kauff & Lutzoni 2002) based on a support threshold of 70. Conflict-free single locus datasets were combined in a concatenated RAxML analysis. Again we used PartitionFinder to identify the optimal number of partitions and substitution models. Accordingly we used two partitions (ITS1/ITS2/mtSSU and 5.8S) with a GTRGAMMA substitution model and generated 500 fast bootstrap replicates.

We also performed Bayesian phylogenetic reconstruction of the ITS dataset using BEAST 2.2.1 (Bouckaert *et al.* 2014). We determined the best site model and partitioning scheme for the ITS dataset (ITS1: GTR+G, 5.8S: TrNef+G, ITS2: GTR+G) with PartitionFinder 1.1.1 according to the BIC criterion and used a relaxed log-normal clock model for each partition. We linked the tree model and subjected it to a Yule tree prior. The MCMC chain was run for 10^7^ generations and every 1000th tree was retained. Convergence of model parameters was assessed using Tracer 1.6 (Rambaut *et al.* 2014) and considered sufficient for ESS values larger than 200. After discarding the first 20% of trees from the posterior tree sample as burn-in, we created a maximum clade credibility tree with TreeAnnotator 2.2.1 included in the BEAST package and randomly selected 100 trees using bash scripts. The maximum clade credibility tree and the set of 100 random trees were used in all further analyses.

### Species delimitation using General Mixed Yule Coalescence

We performed species delimitation with the statistical programming language R (R Development Core Team 2013) using the R package bGMYC (Reid & Carstens 2012). This package provides a Bayesian implementation of the general mixed Yule-coalescence model (Pons *et al.* 2006). Based on a given tree sample, bGMYC models the contribution of between-species divergence (Yule model component) and within-species coalescence events (Coalescent model component) (Pons *et al.* 2006), which give a hypothesis for species delimitation. To account for phylogenetic uncertainty in our dataset we performed all bGMYC analyses on a set of 100 randomly selected trees from the BEAST posterior tree sample (see above). We used the function *bgmyc. multiphylo()* to sample 50 000 steps with a burn-in of 40 000 and the thinning parameter set to 100 for each tree. We then created a co-assignment probability heat map as implemented in the bGMYC function *spec.probmat()*. To determine consensus partitions from the probability heat map, we employed a k-medoid clustering approach as described in Ortiz-Álvarez *et al.* (2015). We used the function *pamk()* in the R package fpc (Hennig 2013) to create groups from the bGMYC co-assignment matrix. *Pamk()* uses a partitioning around medoid (PAM) algorithm, which randomly selects data points from the co-assignment matrix as centres and clusters other data points around those centres according to their distance. The number of clusters is determined by optimum average silhouette width (Hennig 2013).

## Results

### Phylogenetic analyses

**Fig. 1:**
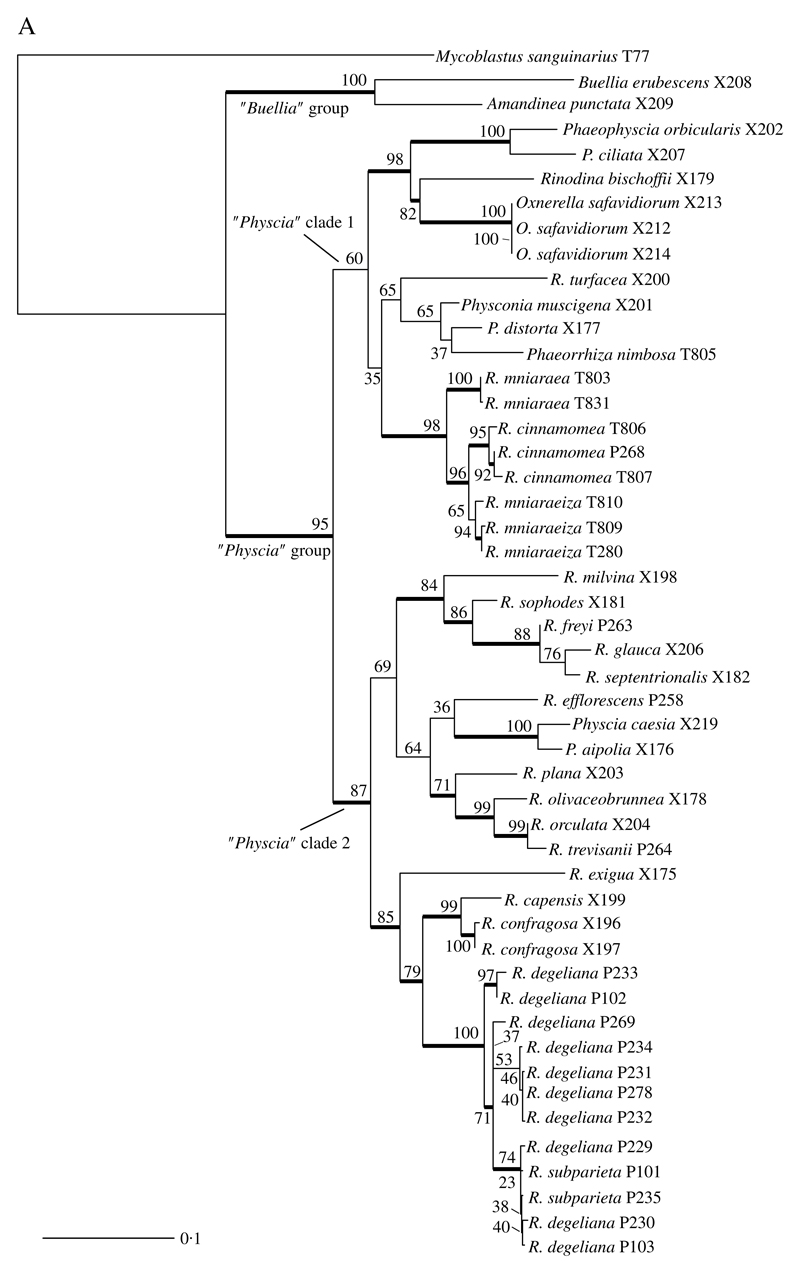

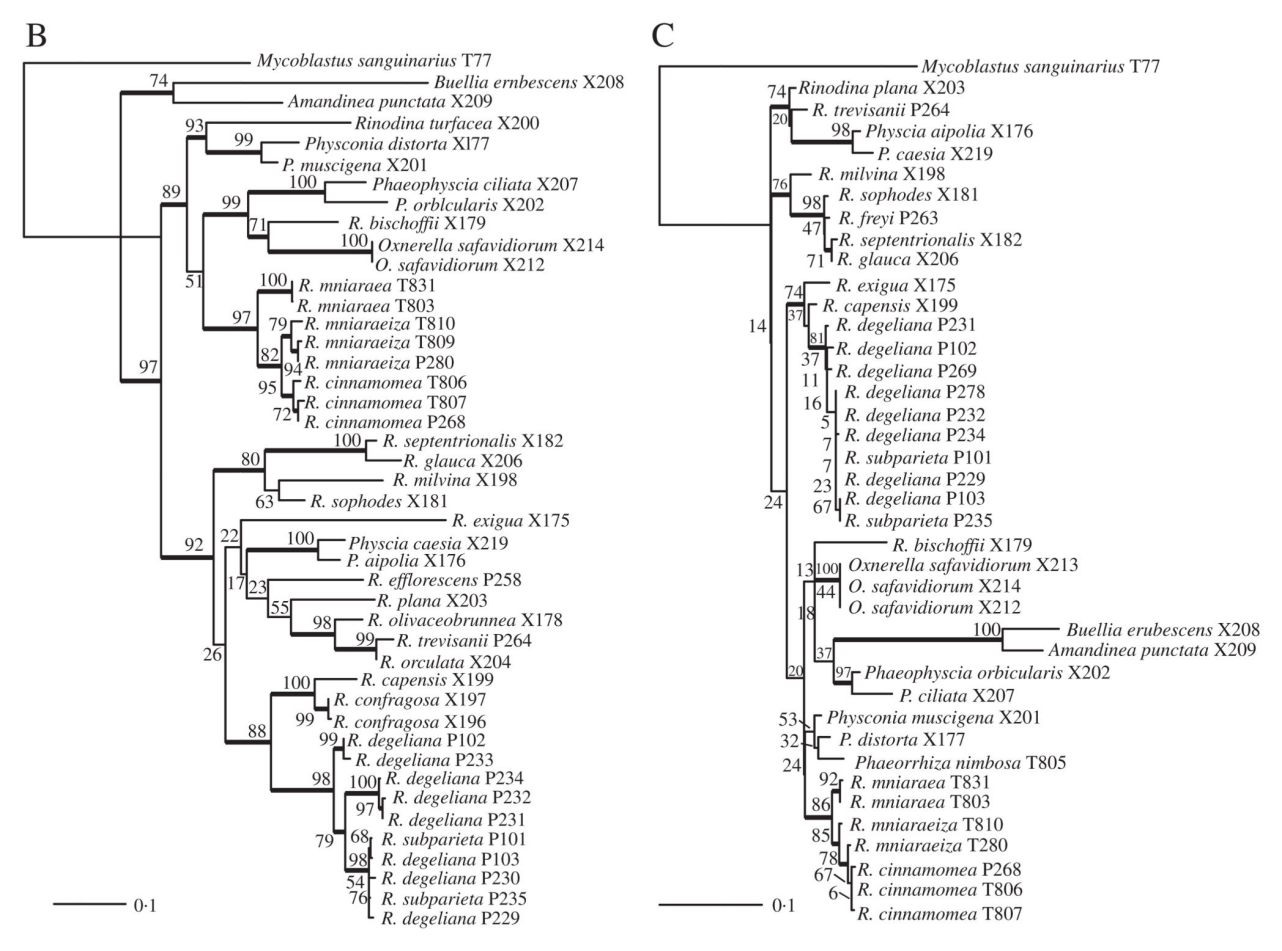
A, maximum-likelihood phylogenetic hypothesis of the concatenated ITS and mtSSU dataset. Branch support is provided as bootstrap values. Branches with high support (bootstrap value ≥70) are indicated in bold. B, maximum-likelihood ITS gene tree. Branch support is provided as bootstrap values. Branches with high support (bootstrap value ≥70) are indicated in bold. C, maximum-likelihood mtSSU gene tree. Branch support is provided as bootstrap values. Branches with high support (bootstrap value ≥70) are indicated in bold. Scales show nucleotide substitutions per site.

We obtained 40 sequences from 24 isolates. Together with the sequences downloaded from GenBank, our dataset consisted of 49 samples and a total of 84 sequences. All sequences used, including those newly published, are summarized in Table 1. Results from our maximum likelihood analyses are depicted in Fig. 1A–C. We identified one conflict between the ITS (Fig. 1B) and mtSSU (Fig. 1C) gene trees coming from specimen P280. As ITS is an important fungal barcode (Schoch *et al.* 2012) and our species delimitations were based on the ITS dataset, we excluded mtSSU sequences of these specimens from the concatenated phylogenetic analysis.

The deepest split in our concatenated maximum likelihood tree (Fig. 1A) corresponded to the “*Buellia*” and “*Physcia*” groups already reported by Nadyeina *et al.* (2010). The “*Buellia*” group, here represented by *Amandinea punctata* (Hoffm.) Coppins & Scheid. and *Buellia erubescens* Arnold was well supported (100% bootstrap support (BS)). The “*Physcia*” group, which received high support (95% BS) was divided into two clades, hereafter referred to as “*Physcia*” clade 1 and 2 (Fig. 1A). “*Physcia*” clade 1 was only moderately supported (60% BS). It contained all samples of *Rinodina mniaraea* s. lat., which formed a well-supported monophyletic group (98% BS). All samples of the varieties *R. mniaraea* var. *mniaraea* and *R. mniaraea* var. *cinnamomea*, respectively, formed well-supported clades (>70% BS). The clade containing *R. mniaraea* var. *mniaraeiza* was supported with 65% BS. Another well-supported (98% BS) lower level clade of “*Physcia*” clade 1 consisted of *Phaeophyscia ciliata* (Hoffm.) Moberg, *P. orbicularis* (Neck.) Moberg, *Rinodina bischoffii* (Hepp) A. Massal. and *Oxnerella safavidiorum.* The latter was resolved as sister to *Rinodina bischoffii* with high support (82% BS). The third subclade of “*Physcia*” clade 1, containing *Rinodina turfacea* (Wahlenb.) Körb., *Physconia muscigena* (Ach.) Poelt, *Phaeorrhiza nimbosa* and *Physconia distorta* (With.) J. R. Laundon, received poor support (65% BS).

“*Physcia*” clade 2 was well supported (87% BS) and contained all samples of *R. degeliana* and *R. subparieta*. They formed a monophyletic group (100% BS) with two subclades, both with high support (71% and 97% BS). Sister to the *R. subparieta*/*degeliana* clade we recovered *R. capensis* Hampe and *R. confragosa* (Ach.) Körb., which form a clade that was preceded by the divergence of *Rinodina exigua* (Ach.) Gray, all also containing atranorin.

Sister to the above group, the “*Physcia*” clade 2 also contained a moderately supported clade (69% BS) consisting of *Rinodina milvina* (Wahlenb.) Th. Fr., *R. glauca* Ropin, *R. septentrionalis* Malme, *R. freyi*, *R. sophodes* (Ach.) A. Massal., *R. efflorescens* Malme, *R. olivaceobrunnea* C. W. Dodge & G. E. Baker, *R. orculata* Poelt & M. Steiner, *R. plana* H. Magn, *R. trevisanii* (Hepp) Körb., *Physcia aipolia* (Ehrh. ex Humb.) Fürnr. and *P. caesia* (Hoffm.) Fürnr., all lacking atranorin except for the *Physcia* species.

**Fig. 2:**
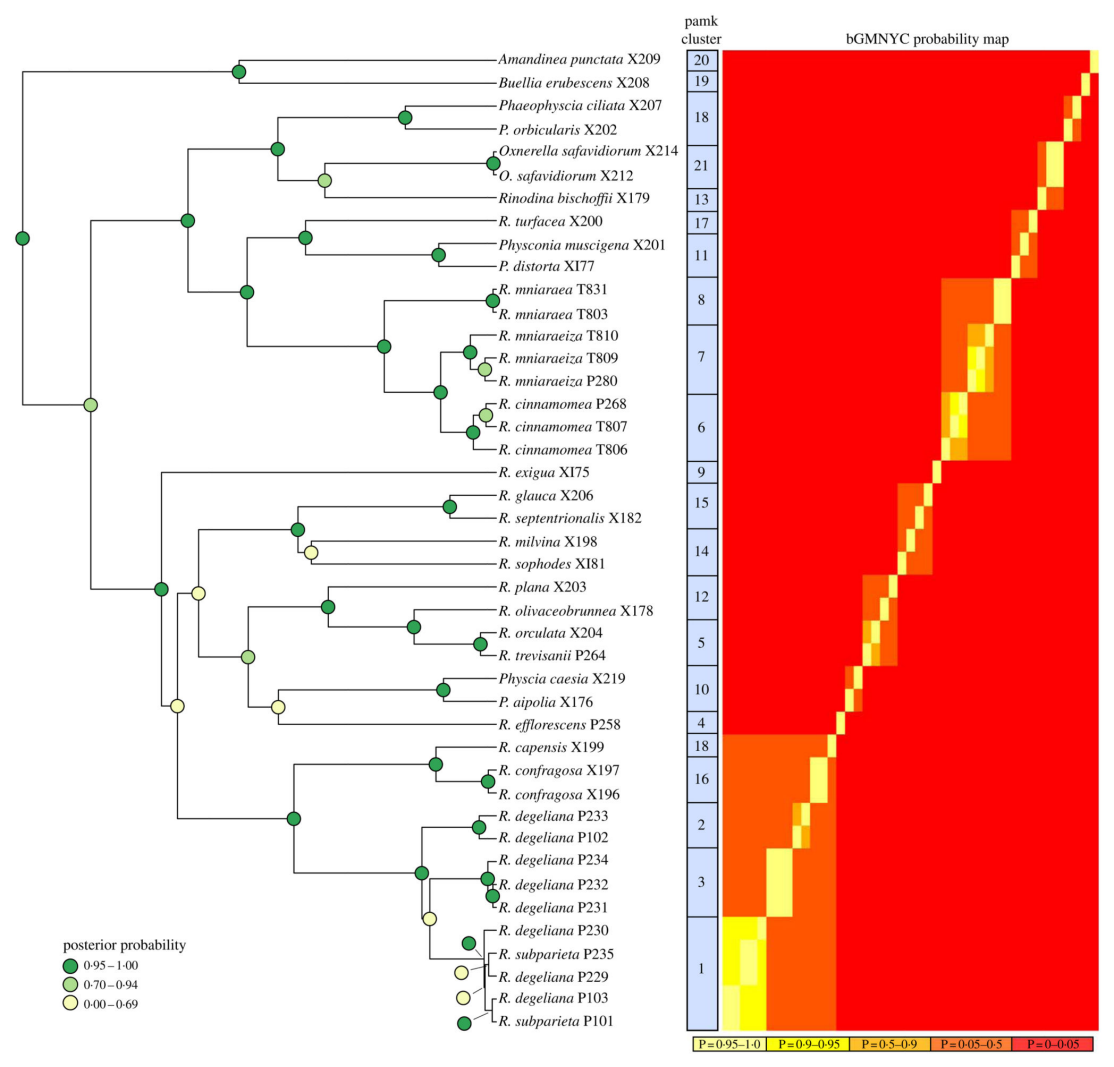
Phylogenetic hypothesis and species delimitation results of the ITS dataset according to the Bayesian General Mixed Yule-Coalesence model. The tree displayed is the maximum clade credibility tree from the BEAST analysis. Node support is provided as coloured circles. Probabilities of the bGMYC probability map are indicative for the chance that tips could be assigned to one species. The pamk clusters refer to grouping based on k-medoids. bGMYC and pamk clustering was based on a tree sample of 100 randomly selected trees from the BEAST posterior distribution of trees.

The BEAST analyses of the ITS dataset led to stable parameter values (ESS > 200) according to Tracer. We only present the BEAST maximum clade credibility tree here (Fig. 2), with posterior probability node support indicated by different colours.

### Species delimitation analysis

Based on the Bayesian mixed Yule-coalescent analysis for species delimitation (Reid & Carstens 2012), we detected significant levels of genetic structure in *Rinodina degeliana* and *R. subparieta*, as well as in *R. mniaraea* s. lat. The pamk clustering approach (see above) detected 21 clusters in the whole dataset. *Rinodina degeliana* and *R. subparieta* samples form a total of three clusters (cluster numbers 1–3; see Fig. 2). The two specimens of *R. subparieta* are assigned to cluster 1, together with three *R. degeliana* samples.

*Rinodina mniaraea* s. lat. also forms three clusters (cluster numbers 6–8; Fig. 2), corresponding to the chemotypes *R. mniaraea* var. *mniaraea*, *R. mniaraea* var. *mniaraeiza* and *R. mniaraea* var. *cinnamomea*.

### Morphological investigation

We undertook an *a posteriori* examination of ascospore size and structure, and soredium size between the *R. subparieta/R. degeliana* clades (Fig. 2) distinguished by the molecular analysis. This was necessarily incomplete due to the absence of ascospores in non-fertile specimens.

The esorediate Japanese collections (P101, P235) had very well-developed thalli and, perhaps for this reason, ascospores were also fully developed (*Physconia*-type) and slightly longer ((20·5–)22·5–24·5(–25·5) × (10·5–) 11·0–11·5(–12·5) μm, *n* = 66, l/w ratio (1·8–) 2·0–2·2(–2·3)) than those found in the type of *R. subparieta* ((19·5–)21·0–23·5(–24·5) × (10·0–)11·0–12·0(–13·0) μm, *n* = 40, l/w ratio (1·7–)1·8–2·0). The *Physconia*-type internal ascospore structure, well-developed torus, and darkly pigmented and strongly ornamented ascospore walls are very similar in all these specimens. It should also be noted that Nylander (1900) measured even larger spores, up to 27 μm long, in the type. In addition, it is notable that the fully developed ascospores of the types of *R. subparieta* and *R. degeliana* are very similarly sized.

**Fig. 3:**
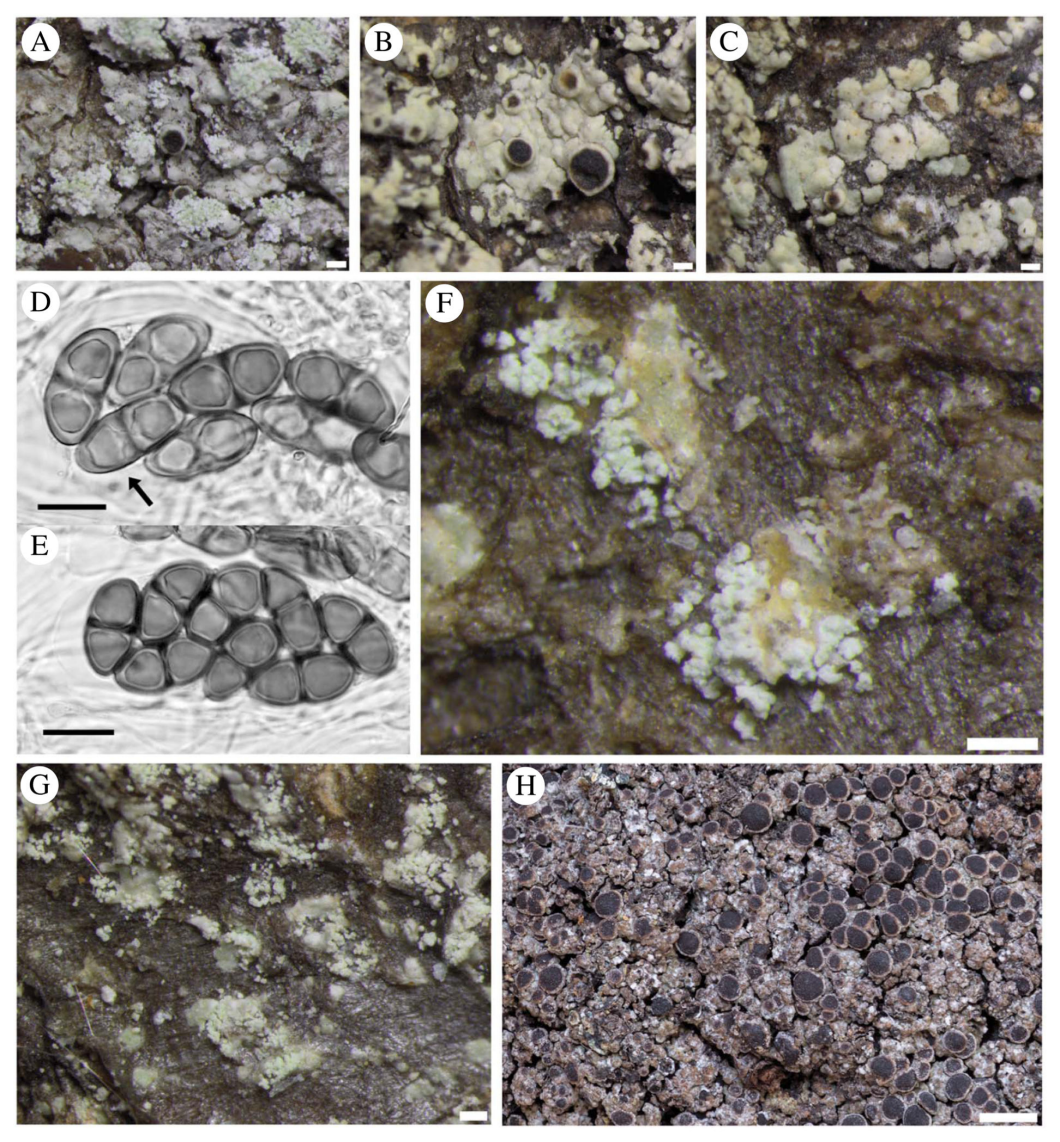
*Rinodina subparieta* (A–G). Fertile sorediate morph (*Lendemer* 43666, GZU); B & C, fertile esorediate morph (*Thor* 28096); D, submature *Physconia*-type ascospores (arrow pointing to *Physcia*-like ascospore with narrow lumin canals and apical wall thickening) (*Tønsberg* 22502, BG); E, mature *Physconia*-type ascospores with broad lumin canals, no apical wall thickening and prominent tori at the septa in various optical sections (*Tønsberg* 22502, BG); F, labriform soralia arising from slightly upturned areole margins (*Tønsberg* 42540); G, sterile morph showing labriform soralia (*Tønsberg* 41921); H, *R. mniaraea* showing typical matt, rugose grey-brownish thallus. Scales: A–C, F & G = 200 μm; D & E = 10 μm; H = 1 mm.

Fertile, sorediate Japanese specimens (representative in molecular study: P103) possessed ascospores that were on average smaller than those of esorediate specimens and apparently always belonged to the *Physconia*-type at maturity, measuring (17·0–)18·5–22·0(–23·5) × 10·0–11·0(–12·5) μm (*n* = 55), l/w ratio (1·5–)1·7–2·0(–2·1). Immature ascospores have broad lumin canals (septal wall thickenings), some with additional apical wall thickening (*Physcia*-like, Fig. 3D). Young ascospores sometimes lose the apical thickening during further development and finally become constricted at the septum. Lumina are then uniformly rounded and thin walled (*Milvina*-like). Measurements for ascospores in specimens with incompletely developed, *Physcia*-type spores tended to be shorter and comparable with those given by Sheard (2010), (15·0–)18·5–19·5 (–23·0) × (8·5–)10·0–11·0(–13·0) μm, for North America. The single fertile eastern North American specimen analyzed (*Lendemer* 43666; P229, Fig. 3A) had poorly formed apothecia with infrequent ascospores that were not measured since they were either too young or too old. Most of its apothecia had abnormally well-developed proper exciples, making them appear lecideine. However, the margins were thalline below, with typically deep cortices filled with atranorin crystals.

Alaskan specimens (P231, P232, P234, pamk cluster 3) were all sorediate and mostly sterile. The single fertile specimen (*Tønsberg* 42694; P231) analyzed from Alaska that yielded mature ascospores was anomalous in that part of the collection lacked atranorin (by TLC and lack of crystals in the cortex), the first such specimen recorded. Some ascospores were of the *Physcia*-type but many clearly trended towards the *Physconia*-type, although retaining some apical wall thickening at maturity. The small sample of ascospores measured from Alaskan material, (18·5–)22·0–24·0(–24·5) × (10·0–)10·5–12·5(–13·0) μm (*n* = 19), l/w ratio (1·7–)1·8–2·1(–2·2), was comparable with the Japanese material cited above. All mature ascospores had a prominent torus but most had unusually light ornamentation. Another fertile, sorediate specimen from Alaska, which was not subject to molecular analysis, had normal chemistry with atranorin and zeorin present.

The Scandinavian and non-Alaskan western North American specimens (pamk cluster 2; Fig. 2) were sterile. However, ascospore sizes for *Rinodina degeliana* quoted by Mayrhofer & Moberg (2002) are comparable with those given above. The very complete type description (Coppins 1983) agrees with our observations of the holo- and isotype except that the ascospores, when fully developed, belong to the *Physconia*-type rather than the *Dirinaria*-type of the protologue, or the *Physcia*-type as reported by Mayrhofer & Moberg (2002) and Sheard (2010). Coppins (1983) reported ascospore measurements of 19–25 × 10–14 μm that were confirmed by our observations (20·5–25·0 × 10·5–13·0 μm, *n* = 14) of the type specimens.

We found that specimens from all clades may possess soredia. Soredia were measured under the compound microscope to an accuracy of 5 μm but showed only a limited variation in size. Specimens from Norway and Washington (P102 and P233 respectively, pamk cluster 2) developed slightly smaller (median 25 μm diam.) soredia compared to 30 μm in other regions of the world ((20–)25–30(–40) μm, *n* = 115). However, this small size difference is accommodated in the 25–75 of the above percentile range and is therefore not meaningful. Soralia develop marginally (somewhat labriform; Fig. 3F & G) on the areoles, and are of similar size in the different clades.

Concerning *Rinodina mniaraea*, Sheard (2010) noted that he searched in vain for morphological, anatomical and ascospore characters to separate the different chemotypes. Numerous collections by Imshaug (MSC) in the southern Rocky Mountains offered the opportunity to test ascospore sizes of different chemotypes from the same collections or from the same localities. Some significant differences were found but they were not consistent between the chemotypes. Therefore, Sheard (2010) was unable to recognize the chemical variants as being taxonomically distinct. Descriptions of the morphology of the taxa based on the chemical variants are found in the following section.

### Taxonomy of *Rinodina subparieta*

**Rinodina subparieta (Nyl.) Zahlbr**.

MycoBank No.: MB816978

*Cat. Lich. Univers*. **7:** 554 (1931).

Basionym: *Lecanora subparieta* Nyl*., Acta Soc. Sci. Fenn*. **26:** 30 (1900); type: Japan, Itchigômé, 1879, *Almquist* (H-Nyl 28856—holotype!).

*Rinodina degeliana* Coppins, *Lichenologist* **15:** 147 (1983); type: Sweden, Lule Lappmark, Kvikkjokk parish, south-east slope of Nammatj, 66°56'N, 17°42'E, alt. 520 m, on *Salix* bark in *Picea* woodlands, 1977, *Coppins* 6238 & *Tibell* (E—holotype!; UPS—isotype!).

(Fig. 3A–G)

*Thallus* epiphloeodal, thin, light grey, areoles to 0·50–0·80 mm wide, subangular or with minutely sublobate margins; areoles isolated around margin, rarely coalescing to become rimose-areolate; surface plane, sometimes becoming rugose, matt or somewhat shining; margin determinate; *prothallus* absent or very thin, sometimes black, visible between marginal areoles; *soredia* whitish (paler than thallus), 20–40 μm diam., arising from underside of slightly upturned areole margins to form labriform soralia (Fig. 3F & G), areoles sometimes dissolving into diffuse soralia, or soredia rarely absent.

*Apothecia* frequently absent, when present typically 1 per areole, becoming narrowly attached, numerous and often contiguous, to 0·50–0·90 mm diam. (Fig. 3A–C); *discs* dark brown or typically black, plane sometimes becoming convex, rarely half-globose; thalline margin 0·05–0·10 mm wide, entire (rarely sorediate in part), persistent or becoming excluded; excipular ring absent or visible when apothecia moistened, narrow and confluent. *Thalline exciple* 60–100 μm wide laterally; cortex 10–20 μm wide; sometimes with epinecral layer 5–10 μm wide; crystals in cortex (atranorin), sometimes in medulla (zeorin); cortical cells to 3·5–6·0 μm, not pigmented, mostly hidden by crystals; algal cells to 6·0–12·0 μm wide; thalline exciple expanding to 90–140 μm below, cortex 25–70 μm deep; *proper exciple* hyaline or very light brown, 5–10 μm wide laterally, expanding to 20–30 μm wide above; *hypothecium* hyaline to very light brown, 70–140 μm deep; *hymenium* (75–)90–120 μm high, not inspersed; *paraphyses* 2·0–2·5 μm wide, strongly conglutinate, apices 3·5–4·5 μm wide, brown capitate, immersed in dispersed pigment forming a red-brown epihymenium; *asci* 60–80 × 15–30 μm. *Ascospores* (rarely 4–)8 per ascus, type A development, *Physcia*- to *Physconia*-type (Fig. 3D & E), (18·0–)20·0–23·5(–25·5) × (10·0–)10·5–12·0(–13·0) μm (*n* = 205), l/w ratio (1·6–)1·8–2·1(–2·2), thickened apical walls during early development and frequently arrested at this stage; torus present; walls mostly darkly pigmented, typically strongly ornamented (×1250).

*Pycnidia* rare, *c*. 0·05 mm diam., immersed in thallus, ostioles brown to black; *pycnoconidia* hyaline, bacilliform 3·5–4·5 × 1·0 μm.

*Chemistry.* Spot tests K+ yellow, C−, P+ bright lemon yellow (very rarely negative). Secondary metabolites: atranorin (very rarely absent) and zeorin. Cortical atranorin typically abundant as indicated by strength of P+ spot test.

*Ecology.* Corticolous, mainly on deciduous trees.

*Notes.* The revised description provided here is based on our studies of *Rinodina degeliana* from Europe (Coppins 1983; Tønsberg 1992; Mayrhofer & Moberg 2002), North America (Sheard 2010) (with the exception of the ascospore dimensions), the type of *R. subparieta* and fertile specimens from Japan collected by GT and TT. *Rinodina subparieta* is a variable species. In addition to having fertile sorediate, fertile esorediate (rare) and non-fertile sorediate morphs (the most frequent state), ascospore size and the development stage attained are also variable. Such ascospore variability is typical of many *Rinodina* species possessing vegetative diaspores (J. W. Sheard, pers. obs.).

The very detailed protologue of *Rinodina degeliana* (Coppins 1983) agrees with our observations of the types of that species. We found only one important difference: that fully developed ascospores belong to the *Physconia*-type, rather than the *Dirinaria*-type or *Physcia*-type as reported by Mayrhofer & Moberg (2002) and Sheard (2010), respectively. Sheard (2010) questioned the distinction between the *Physcia*- and *Physconia*-ascospore types, citing the many *Rinodina* species that have intermediate ascospore types. *Rinodina subparieta* is another such example, with specimens exhibiting the *Physcia*-spore type having arrested ascospore development, a very common feature of the species in Europe, North America and Scandinavia. The ascospore dimensions cited above are from the total of specimens examined in the present study.

The species is easily distinguished from other sorediate *Rinodina* species (such as *R. efflorescens*, *R. griseosoralifera* Coppins, *R. sheardii* Tønsberg and *R. stictica* Sheard & Tønsberg) by its typically light grey areoles with marginal, labriform soralia and whitish soredia, and chemically by the presence of abundant cortical atranorin (P+ strong lemon yellow) and zeorin in its medulla. Very rarely the thallus and the soredia may have a greenish tinge (e.g. specimen in Fig. 3A). We have noted two thalline morphotypes. Most common is the morph with a plane and somewhat shining (waxy) surface, the areoles more or less angular and 0·6–0·8 mm wide, to which the type specimens of *R. degeliana* belong. Very rarely such areoles coalesce to form a rimose-areolate thallus. The second morph, which may be more common in fertile specimens, has smaller areoles, rarely exceeding 0·5 mm in width, with a matt, slightly convex surface and minutely sublobate margins before breaking into soredia. It is notable that the esorediate form of *R. subparieta* has only been found at very high elevations on Honshu, Japan (1960–2550 m), whereas sorediate forms, widely distributed geographically in the Northern Hemisphere, are found at lower elevations, although at up to 1725 m on Honshu (J. W. Sheard, pers. obs.), and only at high elevations in the southern Appalachians (1502–1920 m, *Lendemer* 2528, 44770, 43873 NY).

*Additional fertile, esorediate specimens examined.* **Japan:** *Honshu*: Etchu Prov., Nakasinkawa-gun, 33 km ESE Toyama, Midagahara, along path to Tateyama crater 36°34'N, 137°34'E, alt. 1960–2000 m, subalpine open mixed deciduous *Abies mariesii* forest, on dead, standing, exposed *Abies mariesii*, 1994, *Thor* 12708 (UPS–duplicate in TNS, not seen); Kai Prov. (Yamanashi Pref.), Kofu City, along trail between Odarumi Pass and Mt. Kinpu (Kimpō), old-growth, subalpine forest dominated by *Abies mariesii* and *Tsuga diversifolia*, on *Abies mariesii*, alt. 2552 m, 35°52·163'N, 138°37·930'E (WGS84, ±10 m), 2012, *Thor* 27994 (UPS); Makioka-cho, Yamanashi City, Odarumi pass, near mountain hut, old-growth, subalpine forest dominated by *Abies mariesii,* on *A. mariesii*, alt. 2368 m, 35°52·171'N, 138°40·001'E (WGS84, ±100 m), 2012, *Thor* 28096 (UPS); on *Sorbus* sp., *Thor* 28120 (UPS).

*Selected sorediate specimens examined*. **Japan:** *Hokkaido*: Ishikari Prov., Kamikawa-gun, Kamikawa-cho, 0·5–1·5 km E of Obako Gorge tourist centre, along gravel road just N of Niseicharomappu-gawa stream, open and plane land with deciduous trees, on *Abies sachalinensis*, alt. 720 m, 43°42'N, 143°01'E, 1995, *Thor* 14624 (UPS); Kitami Prov., Esashi-gun, Esashi-cho, 2 km S of Honcho, just SW of road 238, plantation of *Abies sachalinensis* and *Larix gmelinii* var. *japonica* with scattered *Quercus mongolica* var. *grosseserrata*, on *Larix*, alt. 10–20 m, 44°55'N, 142°35'E, 1995, *Thor* 14251 (UPS); Roshiri Island, Oshidomari area, along the path from Rishirihokuroku campsite (2·5 km S of Sakae Town) to Mt. Pon (near the campsite), 45°13'N, 141°13'E, alt. 220 m, old-growth humid forest dominated by *Abies sachalinensis*, corticolous on *Abies sachalinensis*, 1995, *Tønsberg* 22289 (BG); Shari-gun, Shari-cho, Shiretoko National Park, 9 km NE of Utoro Village, along trail around Shiretoko-goko Lakes, 44°07'N, 145°05'E, alt. 260 m, old-growth, mixed deciduous/coniferous forest, on *Betula*, 1995, *Tønsberg* 22797 (BG); Kushiro Prov., Akkeshi-gun, Akkeshi-cho, 44 km E of Kushiro City, S Akkeshiko Lake, just S of road following coast, humid mixed *Abies sachalinensis*/deciduous forest, on *Quercus mongolica* var. *grosseserrata*, alt. 120 m, 42°60'N, 144°57'E, 1995, *Thor* 14486 (UPS); Hamanaka-cho, 55 km E of Kushiro City, 3 km E of Hichiripputo Lake, just S of road following coast, 43°02'N, 145°03'E, alt. 60 m, humid, mixed *Abies sachalinensis*/deciduous forest, corticolous on *Sorbus*, 1995, *Tønsberg* 22936b (BG); Nemuro Prov., Shiretoko Peninsula, Menashi-gun, Rausu-cho, just S of road 334 crossing Shiretoko Peninsula, 4 km WNW of Sakae City, Rausu hot spring, open deciduous forest, on *Alnus* sp., alt. 130 m, 44°02'N, 145°09'E, 1995, *Thor* 14406 (UPS); Teshio Prov., Rumoi-gun, Obira-cho, 21 km ENE of small town Obira at the coast, along the trail from parking area to Tengunotaki Waterfall, 44°04'N, 141°55'E, alt. 50–100 m, dense, old-growth, mixed deciduous forest with scattered *Abies sachalinensis*, corticolous on *Betula*, 1995, *Tønsberg* 21955 (BG); Tokachi Prov., Kato-gun, Kamishihoro-cho, 6 km S of Mikumi Tunnel through Mt. Mikumi-yama, just W of road 273, marsh with *Abies sachalinensis*/deciduous forest, on dead deciduous tree, alt. 680 m, 43°32'N, 143°09'E, 1995, *Thor* 14581 (UPS). *Honshu*: Bizen Prov. (Okayama Pref.), Maniwa-gun, Kawakami-mura, 1–2·3 km N of Hiruzen Research Institute, along road, on exposed tree at roadside, alt. 500–550 m, 35°18'N, 133°38'E, 1994, *Thor* 12212 (UPS); Etchu Prov. (Toyama Pref.), Nakashinkawa-gun (Nakaniikawa-gun), Tateyama-cho, 25 km ESE of Toyama, Bijodaira, path S of Bijodaira bus stop, mixed deciduous/*Cryptomeria japonica* forest, on old deciduous tree, alt. 960–980 m, 36°35'N, 137°28'E, 1994, *Thor* 12664 (UPS); Kai Prov. (Yamanashi Pref.), Makiokacho, Yamanashi City, at road to Odarumi pass, open forest dominated by *Larix*, on *Prunus* sp., alt. 1726 m, 35°49·172'N, 138°38·963'E (WGS84, ±30 m), 2012, *Thor* 28169 (UPS); Shimotsuke Prov. (Tochigi Pref.), 20 km WNW of Nikko, 1 km S of Yumoto Village (36°48'N, 139°26'E), Yudaki Falls, on deciduous tree in open, mixed forest, alt. 1440–1480 m, 1994, *Thor* 12771 (UPS).

### Taxonomy of *Rinodina mniaraea* s. lat.

**Rinodina cinnamomea (Th. Fr.) Räsänen**

*Ann. Acad. Scient. Fenn.* **34**(4): 137 (1931).

Basionym: *Rinodina mniaraea* var. *cinnamomea* Th. Fr., *Nova Acta Regiae Soc. Sci. Upsal.* **3**(3): 228 (1860); type: Norway, Finnmark, Syd-Varanger, Pasvig [Pasvik], 1857, *Th. Fries* (UPS—lectotype! designated by Mayrhofer & Moberg 2002: 72).

*Rinodina mniaraea* var. *chrysopasta* (Lettau) Zahlbr., *Cat. Lich. Univ.* **7**: 533 (1931).—*Rinodina mniaraea* f. *chrysopasta* Lettau, *Hedwigia* **60**: 123 (1918); type: Switzerland, Engadin, am Morteratschgletscher bei Pontresina zwischen Gletscherzunge und dem Gasthaus, 1900 m, 1912, *Lettau* (B—holotype!).

*Thallus* thick, brown, dark brown to copper brown, continuous, rugose, matt.

*Apothecia* abundant, broadly attached, 0·3–1·5 mm diam.; *disc* dark brown to black-brown, rarely pruinose, plane to convex to strongly convex; *thalline exciple* 30–80 μm wide laterally; cortex indistinct; epinecral layer frequent, 10–30 μm wide; *hypothecium* hyaline or light brown at the base, 20–200 μm deep, inspersed with oil droplets; *hymenium* 80–130 μm high; *epihymenium* red-brown to brown. *Ascospores Physcia-*type, 19·0–*27*·*5*–35·0 × 10·0–*12*·*5*–15·0 μm, average l/w ratio 2·2.

*Pycnidia* not observed.

*Chemistry*. Orange to yellow pigment especially in the lower part of the medulla reacting K+ purple to violet; chemotype A: Skyrin, ± variolaric acid, ± *cinnamomea* unknown (5/3/5), ± atranorin; chemotype B: 1-*O*-methylemodin or 8-*O*-methylemodin, ± variolaric acid, ± *cinnamomea* unknown, ± atranorin.

*Ecology and distribution.* On soil, bryophytes and decaying plants often above slightly calcareous and acid bedrock in arctic-alpine habitats. Widespread across the Northern Hemisphere, scattered in more southerly mountains such as the Rocky Mountains, northern Scotland, Alps, Carpathian Mountains, Balkan Peninsula, Anatolian Mountains, Caucasus, Tibet, Himalayas (Mayrhofer & Moberg 2002; Strasser *et al.* 2015).

*Notes.* This species is characterized by a yellow to orange medulla. Chemotype A possesses the anthraquinone skyrin and chemotype B the anthraquinone 1-*O*-methylemodin or 8-*O*-methylemodin. Additional chemical compounds that may occur in different amounts are atranorin, variolaric acid and *cinnamomea* unknown. Based partly on the occurrence of atranorin, Mayrhofer & Moberg (2002) distinguished a total of four chemotypes. The type specimens of *R. mniaraea* var. *cinnamomea* and *R. mniaraea* var. *chrysopasta* belong to chemotype A.

*Selected specimens examined.* **France:** *Dept. Hautes-Alpes*: Dauphine, Umgebung des Col du Lautaret, Epaulement central de Combeynot, 2200–2600 m, 1957, *Poelt* 878 (GZU).—**Germany:** *Bavaria*: Allgäuer Alpen, Höfats-Gufel, 2000 m, 1918, *Lettau* (B).—**Montenegro/Kosovo:** *Prokletije*: Hajla, summit of Hajla mountain, 2403 m, 2010, *Mayrhofer* 464 *& Stešević* (GZU).—**Norway:** *Finnmark*: Mageröy, Skarvaag, 1864, *Th. Fries* (UPS).—**Switzerland:** *Graubünden*: Val Fenga, Ritzenjoch, 2500 m, 1967, *Poelt & Vězda* (BRA).—**China:** *Tibet*: Prov. Xizang, Ningjing Shan Mts., 9 km W Markam (= Gartog), *c*. 4000 m, 1994, *Obermayer* 3798 (GZU).—**Georgia:** *Dzhava*: Kaukasus montes Rachinski khrebet, in vicinitate pagi Tschordi, in monte Tagverula, 2300–2600 m, 1989, *Vašak* (GZU).—**Nepal:** *Mahalangur Himal*: Khumbu, Höhe westlich über Gorak Shep, 5300–5400 m, 1962, *Poelt* L905 (M).—**Pakistan:** *W-Himalaya*: Kaghan Valley, above Saiful-Muluk-Lake, 4070 m, 1990, *Schickhoff* 102/f4 (GZU).—**Turkey:** *Provinz Bursa*: Ulu Dag, 20 km SE von Bursa, 2150 m, 1976, *Kalb* 16068 *& Plöbst* (WIS).

**Rinodina mniaraea (Ach.) Körb**.

*Syst. Lich. Germ.*: 126 (1855).

Basionym: *Lecanora* “*mniaroea*” Ach., *Syn. Lich.*: 339 (1814); type: [Switzerland], Helvetia, *Schleicher* (H-ACH 1136 “91a”—lectotype! designated by Mayrhofer & Moberg 2002).

*Rinodina mniaroeoides* (Nyl.) H. Olivier, *Mém. Soc. Sci. Nat. Cherbourg* **37:** 162 (1909).—*Lecidea mniaroeoides* Nyl., *Flora* **53:** 36 (1870); type: [Russia], Lapponia Kemensis, Muonio, Olostunturi, 1867, *Norrlin* (H-NYL 10657—lectotype! here designated).

*Rinodina amniocola* (Ach.) Körb., *Parerg. Lich.*: 73 (1859).—*Lecanora amniocola* Ach., *Syn. Lich.*: 156 (1814); type: [Switzerland], Helvetia, *Schleicher* (H-ACH 1187—holotype!).

*Rinodina turfacea* var. *nuda* Körb., *Parerg. Lich*.: 72 (1859); type: [Switzerland], Auf der Erde zwischen Moosen bei St. Moritz, *Hepp* [Hepp: Die Flechten Europas Nr. 83] (G—lectotype! here designated).

*Note. Lecanora amniocola* was described in the same publication as *Lecanora mniaroea*, the latter in the appendix. The index was given after the appendix, meaning that it was published at the same time in one issue. We therefore retain the name *mniaraea*, which was used earlier in *Rinodina*.

(Fig. 3H)

*Thallus* usually well developed, grey-brown to brown, continuous, surface usually rugose, rarely developing raised verrucae, matt.

*Apothecia* broadly attached and often contiguous, 0·7–1·1 mm diam.; *disc* dark brown to black-brown, sometimes pruinose especially when young, often becoming convex; *thalline exciple* 70–100 μm wide laterally; cortex 5–10 μm wide; epinecral layer frequent, 5–10 μm wide; *hypothecium* hyaline or light brown in specimens with dark thalli, 80–250 μm deep, inspersed with oil droplets; *hymenium* 80–130 μm high; *epihymenium* red-brown. *Ascospores Physcia*-type 21·0–*27*·*5*–35·0 × 10·0–*12*·5–15·0 μm, average l/w ratio 2·2.

*Pycnidia* immersed in thallus; *pycnoconidia* bacilliform, 4·0 −5·0 × 1·0 μm.

*Chemistry.* Spot tests all negative, secondary metabolite ± variolaric acid.

*Ecology and distribution*. On soil, bryophytes and decaying plants above calcareous and acid bedrock in arctic-alpine habitats, rarely descending into the subalpine forest zone. Widespread across the Northern Hemisphere, scattered in more southerly mountains such as the Rocky Mountains, Sierra Nevada, Pyrenees, Alps, Sudeten, Carpathians, Balkan Peninsula, Caucasus, central Asia, Karakorum and Himalayas (Mayrhofer & Moberg 2002; Mayrhofer *et al.* 2005; Sheard 2010). The record from the South Shetland Islands (Antarctica) by Olech (1989) needs confirmation.

*Note. Rinodina mniaraea* is characterized by the lack of other secondary metabolites apart from variolaric acid.

*Selected specimens examined.* **Germany:** *Allgäuer Alpen*: Obermädli Alpe, *Rehm* (W). *Bayrische Alpen*: Chiemgau, Hochfelln und Hochgern, *Britzelmayr* (PRM).—**Switzerland:** *Bern*: Oberaaralp–Bärenegg, 2400 m, 1920, *Frey* 9075 (G). *Graubünden*: Val Fenga, ad latera montis Heidelberger Spitze, 2500 m, 1967, *Poelt & Vĕzda* (BRA). *Wallis*: Zermatt, Gornergrat, Hochtälligrat, 3130–3150 m, 1961*, Frey* 27168 (G).—**Georgia:** *Caucasus Magnus*: region montis Elbrus, ad latera austro-occidentalia montis Čeget, supra vallem torrentis Baksan, 2800 m, 1980, *Vĕzda* 20659 (PRM).—**Italy:** *South Tyrol*: Hohe Tauern, Venediger Gruppe, Rieserferner-Ahrn Natur Park, SE of Kasern, Röttal, glacier forefield of the Rötkees, 2345 m, 2013, *Bilovitz, Nascimbene, Tutzer, Wallner & Mayrhofer* (GZU).—**Mongolia:** *Archangai Aimak*: Changai, Tarbagatai, 5 km vom Solon-Got-Paß, 2400 m, 1983, *Huneck* (GZU).—**Nepal:** *Zentral-Himalaya*: Langtang-Gebiet, Yala, oberes Langtang, 4830 m, 1986, *Miehe & Miehe* 4595 (GZU).—**Pakistan:** *Karakorum*: Shinghai Gali, 4580 m, 1990, *Miehe & Miehe* 1190 (GZU).—**Turkey:** *Provinz Bursa*: Ulu Dag, 20 km SE von Bursa, 2150 m, 1976, *Kalb* 15879, 16061, 16063 *& Plöbst* (WIS).

**Rinodina mniaraeiza (Nyl.) Arnold**

*Flora* **53:** 469 (1870).

Basionym: *Lecanora* “*mniaroeiza*” Nyl., *Flora* **53:** 33 (1870); type: [Finland], Tavastia australis, Häme, Padasjoki, 1866, *Norrlin* (H-NYL 28734—lectotype! designated by Mayrhofer & Moberg 2002).

*Rinodina mniaraea* f. *biatorina* (Nyl.) Arnold, *Verh. Zool.-Bot. Ges. Wien* **37:** 132 (1887).—*Lecanora turfacea* var. *biatorina* Nyl., *Lich. Scand*.: 151 (1861); type: Norway, Sør-Trøndelag, Dovre, *Schimper* (H-NYL 28717—lectotype! here designated).

*Rinodina hookeri* (Fr.) Dalla Torre & Sarnthein, *Flecht. Tirol* : 206 (1902).—*Parmelia hookeri sensu* Fr. non *Lichen hookeri* Borr. ex Sm., E. Fries, *Lich. Eur.*: 94 (1831); type: [Switzerland], Helvetia, *Schleicher* (UPS—lectotype!, here designated).

*Thallus* light grey, rarely pale brown to brown, continuous, rugose and matt.

*Apothecia* immersed to broadly attached, 0·6–0·9 mm diam.; *thalline exciple* 35–70 μm wide laterally; cortex indistinct; *disc* dark brown to black-brown, without pruina, plane to convex; *hypothecium* 70–250 μm deep, hyaline or light brown to dark brown at the base, inspersed with oil droplets; *hymenium* 85–150 μm high; *epihymenium* light brown. *Ascospores Physcia*-type, 20·0–*25*·*0*–31·5 × 9·0–*11*·*5*–15·0 μm, average l/w ratio 2·15.

*Pycnidia* immersed; *pycnoconidia* bacilliform, 3·8–4·5 × 1·0 μm.

*Chemistry.* Spot test K+ yellow, secondary metabolites atranorin, ± variolaric acid.

*Ecology and distribution.* On soil, bryophytes and decaying plants above calcareous and more rarely acid bedrock in arctic-alpine habitats. Widespread across the Northern Hemisphere, scattered in more southerly mountains such as the Rocky Mountains, Northern Scotland, Alps, Carpathians, Balkan Peninsula, Anatolian mountains and Karakorum (Mayrhofer & Moberg 2002; Strasser *et al.* 2015).

*Note. Rinodina mniaraeiza* is characterized by the presence of atranorin and can be distinguished from *R. mniaraea* and *R. cinnamomea* by the lighter thalli and by slightly smaller ascospores.

*Selected specimens examined.* **Austria:** *Niederösterreich*: Lunz am See, Scheiblingstein, 1500–1600 m, 1995, *Türk* 20285 (GZU).—**Germany:** *Bavaria*: Wettersteingebirge, Hänge nördlich der Alpspitze, *c*. 2000 m, 1966, *Poelt* 13457 (GZU).—**Switzerland:** *Bern*: Grimsel, without date and collector, (BP); Fribourg, Simmental, Kaiseregg, östlicher Vorgipfel ob Waloalp, 2050 m, 1926, *Frey* 10010 (G). *Graubünden*: Unterengadin, Nationalpark, Buffalora Alp, 2140 m, *Frey* 10037 (G). *Obwalden*: Frutt, in den Schratten, 1920 m, 1921, *Frey* 28202 (G).—**Pakistan:** *Karakorum*: Baltistan, Haramosh Range, Alm Pakora SE Ganto La, 3700–3900 m, 1991, *Poelt* K91-171 (GZU). *NW-Himalaya*: Eastern Dersai Plateau, 3950 m, 1991, *Poelt* K91-267 (GZU).—**Turkey:** *Provinz Bursa*: Ulu Dag, 20 km SE von Bursa, 2150 m, 1976, *Kalb* 16048 *& Plöbst* (WIS).

## Discussion

In the present study we have investigated the status of *Rinodina degeliana*, *R. subparieta* and *R. mniaraea* s. lat. By evaluating evidence derived from molecular phylogenetic, chemical and anatomical studies we provide hypotheses about the taxonomy and updated nomenclature for these crustose lichen lineages.

### The status of *Rinodina subparieta* and *R*. *degeliana*

We recovered samples of *Rinodina degeliana* and *R. subparieta* in two well-supported clades in our concatenated maximum likelihood phylogeny (Fig. 1A). According to pamk-clustering based on the bGMYC results (Fig. 2), the two *Rinodina subparieta* samples were assigned to cluster 1 together with samples of *R. degeliana*. However, species delimitation also recovered *Rinodina degeliana* in two additional clusters (pamk cluster 2–3; Fig. 2), indicating multiple previously undiscovered lineages in *R. degeliana*.

The only reliable morphological character to separate *Rinodina degeliana* and *R. subparieta* was the lack of soralia in *R. subparieta*. We took an *a posteriori* approach to identify other possible synapomorphies informed by our phylogenetic hypothesis. Our morphological investigations, however, did not reveal any reliable differences between the *R. degeliana* clades or between *R. degeliana* and *R. subparieta*.

Fertile specimens were rarely observed in the material investigated and occasionally they lacked soralia. Ascospores from this material are variable in size and structure, even when immature and overmature ascospores were excluded from measurement. Such variable ascospores are typical for *Rinodina* species that tend to rely on vegetative reproduction (J. W. Sheard, pers. obs.). Young, not fully pigmented ascospores have *Physcia*-type lumina and mature into darkly pigmented *Physcia*-type ascospores or into *Physconia*-type ascospores also with darkly pigmented walls, a prominent torus and with rather heavily ornamented walls, although the latter character is sometimes less well developed. Asynchronous ascospore development was also frequent among specimens with *Physconia*-type spores; rarely only four ascospores in the ascus reach maturity.

The frequent development of *Physcia*-type ascospores into *Physconia*-type would seem to confirm the opinion of Sheard (2010) that these ascospore types should be regarded as developmental phases of a single ascospore type. Both are accompanied by a distinctive red-brown epihymenium colour in all clades, as are other species possessing these ascospore types (Ropin & Mayrhofer 1993). This coloration is derived from a dispersed red-brown pigment in the epihymenial gelatin together with the dark brown or black capitate pigmentation of the terminal cells of the paraphyses.

Conclusively, *R. degeliana* and *R. subparieta* (and also the different *R. degeliana* clades) were indistinguishable in respect to morphology, anatomy and chemistry. However, molecular results indicate multiple, although in part poorly supported, clades and clusters (Figs. 1A & 2). This could imply multiple species-level lineages in *Rinodina degeliana*. The specimens of *R. subparieta* available to us were nested in one of the *R. degeliana* clades and form a cluster with *R. degeliana* ITS sequences in bGMYC. Our molecular results and the lack of phenotypic characters to separate the two species thus lead us to conclude that *R. subparieta* and *R. degeliana* are conspecific.

We recognize that our interpretation of this species is broad and also includes specimens assigned to possible species-level lineages containing only *R. degeliana* specimens (pamk clusters 2 & 3; Fig. 2). This may represent an oversimplification of the taxonomic status of these lineages. At this point, the formal introduction of new names for any of the three clades would have to be based on molecular evidence only. It is generally possible to describe species based mainly on clade affinity in phylogenetic trees (Leavitt *et al.* 2013a), however we refrain from doing so in the case of *Rinodina degeliana*/*R. subparieta*.

Another solution to this problem, although probably impossible, would be to sequence the type specimens of *R. subparieta* and *R. degeliana*, ascertain which names apply to individual clades and adjust taxonomy accordingly. Since *R. subparieta* is nested in a clade (Fig. 1A) and cluster (Fig. 2) with *R. degeliana* samples and we lack morphological, anatomical and chemical characters to distinguish these lineages, we have been conservative in our interpretation. Further studies, particularly in view of a possible geographical separation, may help reveal the evolutionary history of the clades. Until additional data are available, we propose to use the name *R. subparieta*, which takes priority over *R. degeliana*.

### The status of *R. mniaraea* s. lat

We recovered *Rinodina mniaraea* in three clades (Fig. 1A) and detected three potential species-level lineages in the pamk clustering analysis (Fig. 2). The three clusters correspond to the three previously recognized varieties *Rinodina mniaraea* var. *cinnamomea*, *R. mniaraea* var. *mniaraeiza* and *R. mniaraea* var. *mniaraea.* Searches for morphological and anatomical differences between these lineages have been unsuccessful (Sheard 2010). All form *Lecanora*-type asci with eight *Physcia*-type ascospores, (20·5–)24·5–25·5 (–30·0) × (9·5–)11·5–12·5(–14·5) μm, that have thick apical walls and septal wall thickenings separating the usually unequal-sized cells (Sheard 2010).

Differences between *R. mniaraea* var. *cinnamomea*, *R. mniaraea* var. *mniaraeiza* and *R. mniaraea* var. *mniaraea* are restricted to secondary chemistry (Mayrhofer & Moberg 2002): var. *mniaraea* possesses either no secondary compounds or variolaric acid while var. *mniaraeiza* possesses atranorin and sometimes variolaric acid; var. *cinnamomea* produces the anthraquinones skyrin and 1-*O*-methylemodin or 8-*O*-methylemodin, sometimes with atranorin and variolaric acid or both, and one additional unknown.

Räsänen (1931) had already established *R. cinnamomea* (Th. Fr.) Räsänen and Arnold (1870) transferred *Lecanora* “*mniaroeiza*” described by Nylander (1870) to *Rinodina*. It remains unclear if the lineages discovered by us are truly distinct species. We cannot rule out the alternative hypothesis that *R. mniaraea* varieties all belong to a single species. In the mtSSU gene tree (Fig. 1C) *Rinodina mniaraeiza* does not form a monophyletic group and we thus detected a topological conflict between gene trees. A topological conflict could indicate gene flow. However, at present the size of our molecular dataset of *R. mniararea* s. lat. prevents us from investigating this issue in detail. An alternative explanation could be recent speciation with still incomplete lineage sorting. Additional data are needed to resolve this issue. We failed to find anatomical or morphological characters to separate taxa in *R. mniarea* s. lat. in a situation similar to that of *R. subparieta*/*R. degeliana*. Secondary chemistry, however, is characteristic and corresponded to clade (Fig. 1A) and cluster (Fig. 2) affinity in all *R. mniaraea* s. lat. specimens in our dataset. This is similar to other studies where morphologically indistinguishable taxa were proposed to be chemically distinct species (Lendemer 2012; Leavitt *et al*. 2013b). Our consistent chemical, phylogenetic (but see mtSSU gene tree; Fig. 1C) and species delimitation results lead us to follow Räsänen and Arnold and propose that the varieties of *R. mniaraea* s. lat. should be recognized at the species level.

### The placement of *Oxnerella safavidiorum* in *Physciaceae*

The monotypic genus *Oxnerella* was recently described from the Iranian province Esfahan (Kondratyuk *et al.* 2014b). *Oxnerella safavidiorum* possesses a crustose rusty brown thallus, lecanorine apothecia with a *Biatora*-type ascus and hyaline 1-septate ascospores (Kondratyuk *et al.* 2014b). Kondratyuk *et al.* (2014b) conducted a phylogenetic analysis based on ITS and mtSSU sequences but could not resolve the placement of *Oxnerella*. A possible explanation is that because *Oxnerella* possesses hyaline 1-septate ascospores, Kontratyuk *et al.* (2014b) assumed a position close to *Lecania* in the family *Ramalinaceae*.

Our phylogenetic analysis now resolves *Oxnerella safavidiorum* as sister to *Rinodina bischoffii* with high support (82% BS; Fig. 1A). This placement should be considered tentative as long as fresh material is unavailable to verify sequence identity and rule out contaminations of *Oxnerella* type material. The differences in ascus type and ascospores of *Oxnerella* and *Rinodina* are surprising. A verification of ascus and ascospore characters of *Oxnerella* also has to be postponed until additional material for study is available to us.

## Figures and Tables

**Table 1:** ID, origin, species and used loci of the present molecular studies. IDs indicate laboratory-tracking numbers and also correspond to numbers used in Fig. 1 and the main text. Newly published sequences generated in this study are indicated in bold.

ID	Taxon	Origin	Specimen voucher	ITS+5.8S	mtSSU
X209	*Amandinea punctata*	Ukraine: Donetsk Upland	GZU 000272563	GU553286	GU553306
X208	*Buellia erubescens*	Russia: Komi	KW 63381	GU553289	GU553307
T77	*Mycoblastus sanguinarius*	USA: Montana, Lincoln Co., Laughing Water Creek	*Spribille* 30127-A (GZU)	JF744910	KR017396
X212	*Oxnerella safavidiorum*	Iran	*B. Zarei-Darki* (1216)(KW-L)	KM410153	KM410156
X213	*O. safavidiorum*	Iran	*B. Zarei-Darki* (2474)(KW-L)	–	KM410155
X214	*O. safavidiorum*	Iran	*B. Zarei-Darki* (3199)(KW-L)	KM410151	KM410154
X207	*Phaeophyscia ciliata*	unknown	*Himelbrant* K-04-23	EF582752	EF582803
X202	*P. orbicularis*	Belgium: District Mosan	*Ertz* 7648 (BR)	JQ301694	DQ912289
T805	*Phaeorrhiza nimbosa*	Montenegro: Hajla	*Mayrhofer* s.n. (GZU)	–	**KX015707**
X176	*Physcia aipolia*	Spain	MAF 7464	AY449723	AY464069
X219	*P. caesia*	unknown	*Tehler* 7886	AF224388	EU682140
X177	*Physconia distorta*	Spain: Caceres	MAF-lich 9896	DQ862482	AY464068
X201	*P. muscigena*	Canada: Nunavut	AFTOL-ID 220	JQ301696	DQ912291
X179	*Rinodina bischoffii*	Ukraine: Donetsk Upland	KW 63380	GU553291	GU553311
X199	*R. capensis*	Austria: Steiermark, Schladminger Tauern	*Mayrhofer, Harutyunyan & Nadyeina* s.n. (GZU)	GU553293	GU553313
P268	*R. cinnamomea*	Austria: Tirol, Ötztaler Alps	*Mayrhofer* 20801 (GZU)	**KX015685**	**KX015704**
T806	*R. cinnamomea*	USA: Montana, Lincoln Co., Bluebird Basin	*Spribille* 19893 (GZU)	**KX015688**	**KX015708**
T807	*R. cinnamomea*	Canada: British Columbia, Selkirk Mtns., Silvercup Ridge	*Spribille* 20101 (GZU)	**KX015689**	**KX015709**
X196	*R. confragosa*	Austria: Styria, Schladming	*Obermayer* 09091 (GZU)	DQ849297	–
X197	*R. confragosa*	unknown	unknown; sequence published in Grube & Arup (2001)	AF250808	–
P102	*R. degeliana*	Norway: Nordland, Hemnes	*Tønsberg* 41921	**KX015674**	**KX015695**
P103	*R. degeliana*	Japan: Yamanashi, Honshu	*G. Thor* 28169	**KX015675**	**KX015694**
P229	*R. degeliana*	USA: New York, Hamilton County	*Lendemer* 43666	**KX015676**	**KX015696**
P230	*R. degeliana*	Canada: New Brunswick, Sunbury County	*Clayden* 24048 (NBM)	**KX015677**	–
P231	*R. degeliana*	USA: Alaska, Katmai	*Tønsberg* 42694	**KX015678**	**KX015697**
P232	*R. degeliana*	USA: Alaska, Katmai	*Tønsberg* 42573	**KX015679**	**KX015698**
P233	*R. degeliana*	USA: Washington	*Tønsberg* 42540	**KX015680**	–
P234	*R. degeliana*	USA: Alaska, Katmai	*Tønsberg* 42631	**KX015681**	**KX015699**
P269	*R. degeliana*	Canada: British Columbia, Clearwater River	*Spribille & Goward* 11.09.2014	–	**KX015705**
P278	*R. degeliana*	USA: Alaska, Kenai Peninsula	*Tønsberg* 44223	–	**KX015712**
P258	*R. efflorescens*	Czech Republic: Southern Bohemia, distr. Česky Krumlov	*Maliček* 5462 (duplicate)	**KX015683**	–
X175	*R. exigua*	Sweden: Oestergotland	*Mayrhofer* s.n. (GZU)	GU553294	GU553314
P263	*R. freyi*	Austria: Steiermark, Oststeirisches Riedelland, Laßnitzhöhe	*Obermayer* 12318	–	**KX015701**
X206	*R. glauca*	Austria: Steiermark, Schladminger Tauern	*Mayrhofer, Harutyunyan & Nadyeina* s.n. (GZU)	GU553295	GU553315
X198	*R. milvina*	Ukraine: Donetsk Upland	KW 63379	GU553299	GU553317
T803	*R. mniaraea*	USA: Idaho, Boundary Co., Mt. Roothaan	*Spribille* 15242 (GZU)	**KX015687**	**KX015706**
T831	*R. mniaraea*	USA: Montana, Lincoln Co., Wolverine/Bluebird Divide	*Spribille* 20391 (GZU)	**KX015692**	**KX015711**
P280	*R. mniaraeiza*	Austria: Tirol, Ötztaler Alps	*Mayrhofer* 20802 (GZU)	**KX015686**	**KX015713**
T809	*R. mniaraeiza*	Canada: British Columbia, Goat Range, Dennis Creek	*V. Wagner* 18.07.06/1 (GZU)	**KX015690**	–
T810	*R. mniaraeiza*	Canada: British Columbia, Selkirk Mtns., Silvercup Ridge	*V. Wagner* 15.07.06/1 (GZU)	**KX015691**	**KX015710**
X178	*R. olivaceobrunnea*	Antarctica: Lagoon Island	*Romeike* 2.090300 (GOET)	AF540547	–
X204	*R. orculata*	Austria: Steiermark, Zirbitzkogel	*Mayrhofer* 15754 (GZU)	DQ849309	–
X203	*R. plana*	unknown	unknown; sequences published in Grube & Arup (2001)	AF250812	AY143425
X182	*R. septentrionalis*	Russia: Komi	GZU 000272561	GU553303	GU553320
X181	*R. sophodes*	Austria: Steiermark, Schladminger Tauern	*Mayrhofer, Harutyunyan & Nadyeina* s.n. (GZU)	GU553304	GU553321
P101	*R. subparieta*	Japan: Yamanashi, Honshu	*G. Thor* 28096	**KX015673**	**KX015693**
P235	*R. subparieta*	Japan: Yamanashi, Honshu	*G. Thor* 28120	**KX015682**	**KX015700**
P264	*R. trevisanii*	Mongolia: Khangai	*de Bruyn* s.n. 2011 (GZU)	**KX015684**	**KX015702**
X200	*R. turfacea*	Sweden	*Moberg* 10422	AF224362	–
